# Fear Extinction and Predictive Trait-Like Inter-Individual Differences in Rats Lacking the Serotonin Transporter

**DOI:** 10.3390/ijms22137088

**Published:** 2021-06-30

**Authors:** Maria Willadsen, Metin Uengoer, Anna Sługocka, Rainer K.W. Schwarting, Judith R. Homberg, Markus Wöhr

**Affiliations:** 1 Behavioral Neuroscience, Experimental and Biological Psychology, Faculty of Psychology, Philipps-University of Marburg, Gutenberg-Str. 18, D-35032 Marburg, Germany; willadsm@staff.uni-marburg.de (M.W.); schwarti@staff.uni-marburg.de (R.K.W.S.); 2 Associative Learning, Experimental and Biological Psychology, Faculty of Psychology, Philipps-University of Marburg, Gutenberg-Str. 18, D-35032 Marburg, Germany; uengoer@staff.uni-marburg.de; 3 Department for Experimental Medicine, Faculty of Medical Sciences in Katowice, Medical University of Silesia, Medyków 4, 40-752 Katowice, Poland; anna.slugocka@sum.edu.pl; 4 Department of Physiology, Faculty of Medical Sciences in Katowice, Medical University of Silesia, Medyków 18, 40-752 Katowice, Poland; 5 Center for Mind, Brain and Behavior, Philipps-University of Marburg, Hans-Meerwein-Str. 6, D-35032 Marburg, Germany; 6 Department of Cognitive Neuroscience, Donders Institute for Brain, Cognition and Behaviour, Radboud University Nijmegen Medical Centre, Kapittelweg 29, 6525 EN Nijmegen, The Netherlands; Judith.Homberg@radboudumc.nl; 7 KU Leuven, Faculty of Psychology and Educational Sciences, Research Unit Brain and Cognition, Laboratory of Biological Psychology, Social and Affective Neuroscience Research Group, B-3000 Leuven, Belgium; 8 KU Leuven, Leuven Brain Institute, B-3000 Leuven, Belgium

**Keywords:** fear conditioning, freezing, ultrasonic vocalizations, alarm calls, novelty-seeking, anxiety, cognition, pain, SERT, 5-HTT

## Abstract

Anxiety disorders are associated with a failure to sufficiently extinguish fear memories. The serotonergic system (5-hydroxytryptamine, 5-HT) with the 5-HT transporter (5-HTT, SERT) is strongly implicated in the regulation of anxiety and fear. In the present study, we examined the effects of SERT deficiency on fear extinction in a differential fear conditioning paradigm in male and female rats. Fear-related behavior displayed during acquisition, extinction, and recovery, was measured through quantification of immobility and alarm 22-kHz ultrasonic vocalizations (USV). Trait-like inter-individual differences in novelty-seeking, anxiety-related behavior, habituation learning, cognitive performance, and pain sensitivity were examined for their predictive value in forecasting fear extinction. Our results show that SERT deficiency strongly affected the emission of 22-kHz USV during differential fear conditioning. During acquisition, extinction, and recovery, SERT deficiency consistently led to a reduction in 22-kHz USV emission. While SERT deficiency did not affect immobility during acquisition, genotype differences started to emerge during extinction, and during recovery rats lacking SERT showed higher levels of immobility than wildtype littermate controls. Recovery was reflected in increased levels of immobility but not 22-kHz USV emission. Prominent sex differences were evident. Among several measures for trait-like inter-individual differences, anxiety-related behavior had the best predictive quality.

## 1. Introduction

Excessive anxiety and fear are hallmarks of a number of neuropsychiatric disorders, most notably anxiety disorders, including phobias and post-traumatic stress disorder (PTSD) [1]. It is thought that such neuropsychiatric disorders are associated with a failure to sufficiently extinguish fear memories [2]. Fear extinction is the inhibition of conditioned fear responses that is normally seen as a consequence of repeated exposure to a conditioned stimulus (CS) in the absence of an aversive unconditioned stimulus (UCS) [3]. It is thus a key component of the widely applied exposure therapy in the treatment of excessive anxiety and fear [4]. Limiting the efficacy of exposure therapy, however, stress and other environmental factors can inhibit fear extinction [5]. Moreover, there is a large variability between individuals and personality traits appear to play a prominent modulatory role [6]. Although recent efforts helped to dissect neurobiological mechanisms underlying fear extinction [7], little is still known about neurobiological factors associated with personality traits modulating fear extinction. 

A prime candidate for explaining a significant proportion of the inter-individual differences seen during fear extinction is the serotonergic system (5-hydroxytryptamine, 5-HT). The 5-HT system fulfills a wide variety of functions and is strongly implicated in the regulation of anxiety and fear [8,9,10]. For instance, it is closely associated with anxiety disorders, including PTSD [11,12]. A key component of the 5-HT system is the 5-HT transporter (5-HTT, SERT), which regulates 5-HT availability in the synaptic cleft and terminates 5-HT signaling through reuptake of 5-HT into the presynaptic terminal [13].

In humans, the polymorphic region in the promoter of the SERT gene *SLC6A4* (5-HTTLPR) leads to the formation of two major variations, a short and a long allelic variant [14]. The short allelic variant leads to reduced transcription and altered function of SERT, translating into elevated levels of neuroticism, which includes higher anxiety levels [15], and increased acquisition [16] followed by reduced extinction [17] of fear. At the neurobiological level, such traits are paralleled by anatomical and functional correlates in multiple brain regions, including stronger amygdala activation in response to fearful stimuli [18]. Together, this contributes to a greater risk for suffering from PTSD after stressful life events [19]. 

By partially or fully reducing SERT expression through genetic modification, the consequences of limited SERT availability can be modelled in mice and rats [20,21]. In rats, the complete absence of SERT leads to a prominent increase in basal extracellular 5-HT levels and unresponsiveness to the selective 5-HT reuptake inhibitor citalopram [21]. Similar to human carriers of the short allelic variant, anxiety-related behavior is enhanced in rats lacking SERT. This is observable in unconditioned tasks, such as open field, elevated plus maze, light-dark test, and novelty suppressed feeding [22,23,24,25,26].

Fear extinction can be studied in rats [27] and SERT availability was found to play a modulatory role [28]. While acquisition of fear-related behavior towards threat signaling stimuli appears not to be affected in the majority of studies, the ability to extinguish fear-related behavior was repeatedly found to be impaired [23,25,29,30,31,32,33,34,35]. Furthermore, lack of SERT impedes extinction recall by means of recovery of formerly extinguished fear-related immobility [32,34]. Evidence for a conserved role of SERT in fear extinction was also obtained in mouse studies [36]. 

We have recently applied a fear conditioning paradigm in rats lacking SERT and obtained evidence for strong effects on fear-related behavior by measuring alarm calls in addition to immobility [37]. Rats emit whistle-like calls in the ultrasonic range, so called ultrasonic vocalizations (USV) [38,39]. In aversive situations, long and low-frequency 22-kHz USV occur. Under natural conditions, they can be observed during aggressive interactions with conspecifics [40,41] and exposures to predators or their odors [42,43]. Standardized procedures to evoke them in the laboratory include the administration of air puffs [44,45,46], acoustic startle stimuli [47,48], and electric shocks [49,50]. The emission of 22-kHz USV is believed to reflect a negative affective state akin to anxiety and fear. In fact, 22-kHz USV serve as additional measures in fear conditioning experiments because they allow to reveal effects of experimental manipulations that the standard measure immobility fails to capture [51]. For instance, early life stressors, such as prenatal exposure to the viral mimic polyI:C, were found to enhance 22-kHz USV emission in absence of overt behavioral differences [52]. In our most recent study on the effects of SERT availability, we found that rats lacking SERT emitted fewer 22-kHz USV than controls [37]. This effect was seen in absence of overt behavioral differences despite a detailed behavioral analysis and was found to be more prominent in females than in males [37].

This genotype effect is particularly relevant in the context of personality traits modulating fear extinction because the emission of 22-kHz USV is characterized by robust inter-individual differences. For instance, rats characterized by high levels of trait anxiety emit more 22-kHz USV when challenged with tone-shock pairings during fear conditioning [53]. Moreover, factors known to shape trait-like inter-individual differences were repeatedly associated with alterations in 22-kHz USV emission. This includes prenatal immune activation [52], maternal neglect [54], and juvenile stress exposure [55]. 

Together, fear extinction is affected by various factors, resulting in substantial variability between individuals. In the present study, we aimed at identifying trait-like inter-individual differences driving a significant proportion of the variability between individuals seen during fear extinction. To this aim, we examined the effects of SERT deficiency on fear extinction in a differential fear conditioning paradigm in male and female rats. During differential fear conditioning, one CS was repeatedly paired with electric foot shocks (CS+) but not the other (CS-). Fear-related behavior displayed during acquisition, extinction, and recovery, was measured through quantification of 22-kHz USV emission and immobility. Trait-like inter-individual differences in novelty-seeking, anxiety-related behavior, habituation learning, cognitive performance, and pain sensitivity were examined for their predictive value in forecasting fear extinction. 

## 2. Results

### 2.1. Body Weight

**Figure 1:**
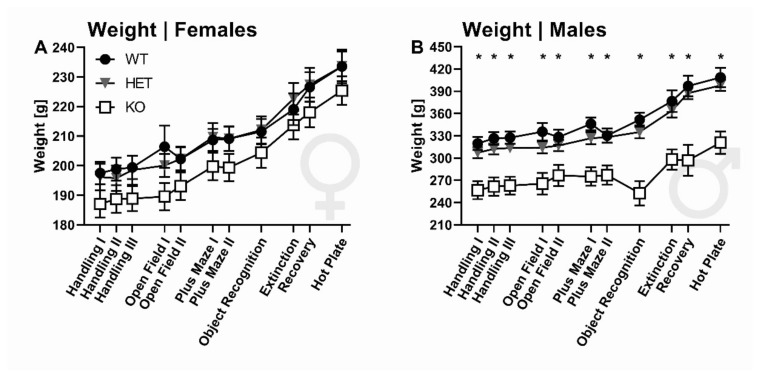
Body weight. Effects of SERT deficiency on body weight across different testing procedures for (**A**) female and (**B**) male SERT^+/+^ (black circles), SERT^+/−^ (grey triangle), and SERT^−/−^ (white square) rats. *N* = 44 female rats (15 +/+, 15 +/−, 14 −/−), N = 43 male rats (14 +/+, 15 +/−, 14 −/−). Data are presented as mean ± SEM. * *p* < 0.05 effect of genotype, as compared to SERT^+/−^ and SERT^+/+^ rats.

SERT deficiency affected body weight in a sex-dependent manner (G: F_2,87_ = 14.093, *p* < 0.001; S: F_1,87_ = 262.820, *p* < 0.001; GxS: F_2,87_ = 9.216, *p* < 0.001). While no prominent genotype differences were evident in females (all *p* > 0.05, Figure 1A), SERT deficiency affected body weight in males, with male SERT^−/−^ rats showing consistently lower body weights than SERT^+/−^ and SERT^+/+^ littermates (all *p* < 0.001, Figure 1B).

### 2.2. Differential Fear Conditioning 

#### 2.2.1. 22-kHz USV Prevalence

**Figure 2:**
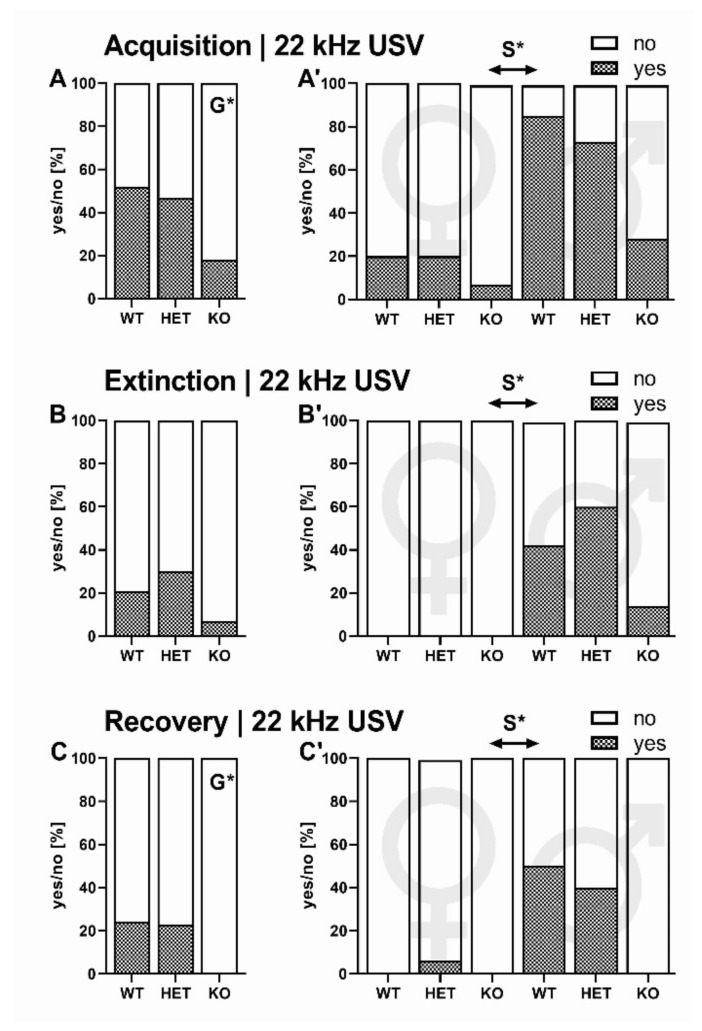
22-kHz USV prevalence. Effects of SERT deficiency on the prevalence of 22-kHz emission (yes—checkered bar; no—transparent bar) during acquisition (**A**,**A’**), extinction (**B**,**B’**), and recovery (**C**,**C’**). Prevalence of 22-kHz USV is shown for SERT^+/+^, SERT^+/−^, and SERT^−/−^ rats with sexes pooled (**A**,**B**,**C**) and separated by sex (**A’**,**B’**,**C’**), with females on the left and males on the right side of the panel. *N* = 44 female rats (15 +/+, 15 +/−, 14 −/−), N = 43 male rats (14 +/+, 15 +/−, 14 −/−). Data are presented as mean ± SEM. G* *p* < 0.05 effect of genotype, S* *p* < 0.05 effect of sex.

When exposed to tone-shock pairings during the acquisition phase of the first day of differential fear conditioning, the emission of 22-kHz USV was strongly affected by genotype (G: chi^2^_2_ = 7.970, *p* = 0.019) and sex (S: chi^2^_2_ = 20.077, *p* < 0.001). While 52% (*N* = 15 out of *N* = 29) of SERT^+/+^ and 47 % (*N* = 14 out of *N* = 30) of SERT^+/−^ rats emitted 22-kHz USV during acquisition, 22-kHz USV emission rates were low in SERT^−/−^ littermates and only 18% (*N* = 5 out of *N* = 28) vocalized (Figure 2A). Genotype differences appear to be driven by male rats. With 63 % (*N* = 27 out of *N* = 43) the majority of male rats emitted 22-kHz USV, whereas only 16 % (*N* = 7 out of *N* = 44) of females did (Figure 2A).

When challenged with CS presentations in another context during extinction on the second day, the emission of 22-kHz USV tended to differ between genotypes (G: chi^2^_2_ = 4.849, *p* = 0.089) and was strongly affected by sex (S: chi^2^_2_ = 21.620, *p* < 0.001). During extinction, 21% (*N* = 6 out of *N* = 29) of SERT^+/+^ and 30% (*N* = 9 out of *N* = 30) of SERT^+/−^ rats but only 7% (*N* = 2 out of *N* = 28) of SERT^−/−^ littermates emitted 22-kHz USV (Figure 2B). Again, genotype effects were driven by male rats. With 40% (*N* = 17 out of *N* = 43) a large number of male rats emitted 22-kHz USV, whereas no female did (*N* = 0 out of *N* = 44, Figure 2B’).

Seven days after extinction training, rats were reintroduced to the extinction context. Emission of 22-kHz USV was affected by genotype (G: chi^2^_2_ = 7.925, *p* = 0.019) and sex (S: chi^2^_2_ = 12.591, *p* < 0.001). While 24% (*N* = 7 out of *N* = 29) of SERT^+/+^ and 23% (*N* = 7 out of *N* = 30) of SERT^+/−^ rats emitted 22-kHz USV during recovery, no 22-kHz USV were detected in SERT^−/−^ littermates (*N* = 0 out of *N* = 28, Figure 2C). Similar to acquisition and extinction, genotype effects were driven by male rats. When split into sexes, 30% (*N* = 13 out of *N* = 43) of male rats emitted 22-kHz USV, whereas only 2% (*N* = 1 out of *N* = 44) of females did (Figure 2C).

#### 2.2.2. Overall Immobility and 22-kHz USV Total Calling Time

**Figure 3:**
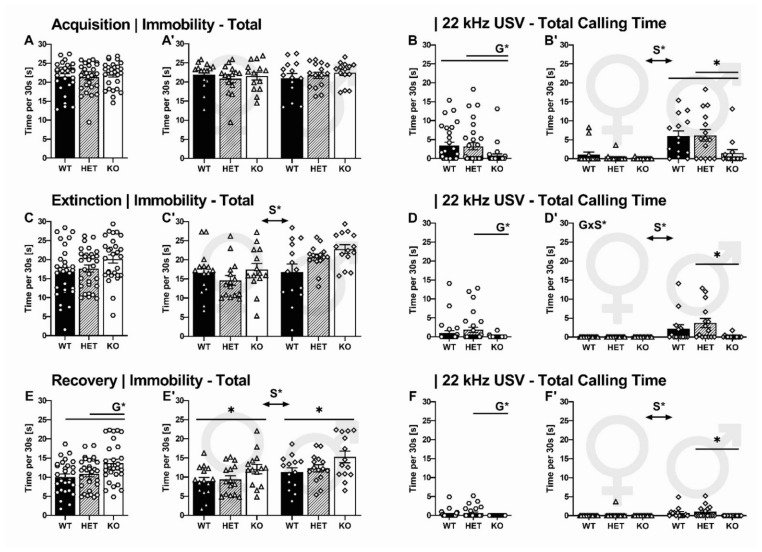
Overall immobility and 22-kHz total calling time. Effects of SERT deficiency on overall immobility and 22-kHz USV total calling time during acquisition (**A**,**B**), extinction (**C**,**D**), and recovery (**E**,**F**). Time spent immobile is shown for SERT^+/+^ (black bar), SERT^+/−^ (striped bar), and SERT^−/−^ (white bar) rats with sexes pooled (**A,C,E**) and separated by sex (**A,C,E**), with females on the left and males on the right side of the panel. Time spent calling 22-kHz USV is shown for rats with sexes pooled (B,D,F) and separated by sex (**B,D,F**), with females on the left and males on the right side of the panel. *N* = 44 female rats (15 +/+, 15 +/−, 14 −/−), N = 43 male rats (14 +/+, 15 +/−, 14 −/−). Data are presented as mean ± SEM. G* *p* < 0.05 effect of genotype, with lines indicating significant post-hoc comparison between genotypes. S* *p* < 0.05 effect of sex. * *p* < 0.05 for subgroup comparison.

During acquisition, the overall time spent immobile was high in all experimental conditions irrespective of genotype (G: F_2,87_ = 0.265, *p* = 0.768, Figure 3A) and sex (S: F_1,87_ = 0.135, *p* = 0.714, GxS: F_2,87_ = 0.563, *p* = 0.572, Figure 3A’). Consistent with 22-kHz USV prevalence, however, the time spent emitting 22-kHz USV during acquisition was affected by genotype (G: F_2,87_ = 4.688, *p* = 0.012) and sex (S: F_1,87_ = 25.538, *p* < 0.001, GxS: F_2,87_ = 2.818, *p* = 0.066). SERT^−/−^ rats spent less time calling compared to their SERT^+/−^ and SERT^+/+^ littermates (*p* = 0.014 and *p* = 0.008, respectively; Figure 3B). The genotype effect was driven by males, which spent considerably more time emitting 22-kHz USV than their female conspecifics (Figure 3B).

During extinction on the second day, a genotype difference in the overall time spent immobile tended to emerge (G: F_2,87_ = 2.989, *p* = 0.056, Figure 3C). Moreover, immobility was affected by sex (S: F_1,87_ = 10.758, *p* = 0.002, GxS: F_2,87_ = 2.471, *p* = 0.091, Figure 3C), with female rats showing less immobility than males. Similarly, time spent calling during extinction was affected by genotype (G: F_2_ = 4.265, *p* = 0.017, Figure 3D) and sex (S: F_1,87_ = 15.040, *p* < 0.001, Figure 3D). Furthermore, there was an interaction between genotype and sex (GxS: F_2,87_ = 4.265, *p* = 0.017, Figure 3D). SERT^−/−^ rats spent less time calling compared to their SERT^+/−^ but not SERT^+/+^ littermates (*p* = 0.010 and *p* = 0.157, respectively). As during acquisition, the genotype effect was driven by males because female rats did not emit 22-kHz USV and therefore differed in time spent calling compared to their male conspecifics.

During recovery, immobility was affected by genotype (G: F_2,87_ = 5.354, *p* = 0.007, Figure 3E) and sex (S: F_1,87_ = 9.115, *p* = 0.003, GxS: F_2,87_ = 0.066, *p* = 0.936, Figure 3E’). SERT^−/−^ rats spent more time immobile compared to their SERT^+/−^ and SERT^+/+^ littermates (*p* = 0.015 and *p* = 0.002, respectively). As during extinction, female rats displayed lower levels of immobility in comparison to their male conspecifics. Despite this sex difference, however, the genotype effect was robust and SERT^−/−^ rats of both sexes spent more time immobile compared to their SERT^+/+^ littermates (*p* = 0.044 and *p* = 0.027, respectively). Parallel to 22-kHz USV emission during acquisition and extinction, time spent emitting 22-kHz USV during recovery was affected by genotype (G: F_2,87_ = 3.452, *p* = 0.036, Figure 3F) and sex (S: F_1,87_ = 6.493, *p* = 0.013, GxS: F_2,87_ = 1.602, *p* = 0.208, Figure 3F). With SERT^−/−^ rats lacking 22-kHz USV during recovery, time spent calling differed from their SERT^+/−^ but not SERT^+/+^ littermates (*p* = 0.010 and *p* = 0.151). Again, the genotype effect was driven by males because female rats spent less time calling than their male conspecifics.

#### 2.2.3. 22-kHz USV: Temporal Emission Pattern

**Figure 4:**
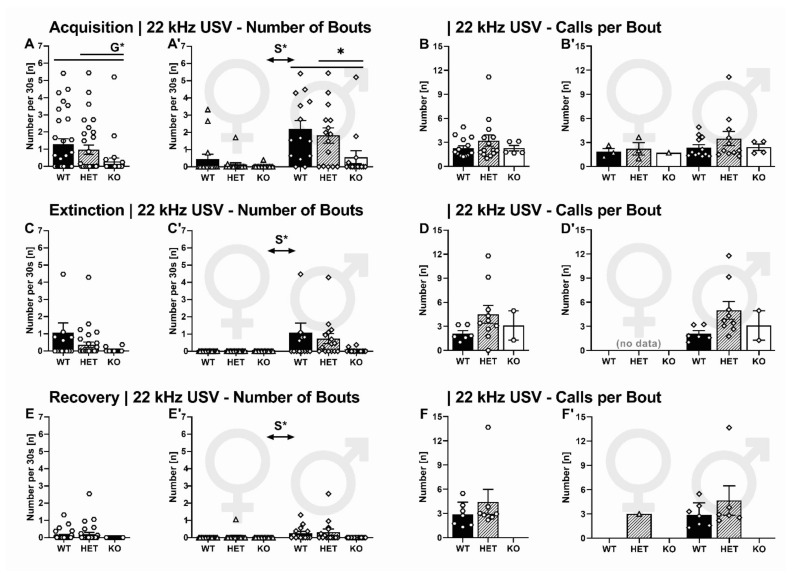
Temporal structure of 22-kHz USV emission. Effects of SERT deficiency on the temporal 22-kHz USV emission pattern during acquisition (**A**–**B’**), extinction (**C**–**D**’), and recovery (**E**–**F’**). The number of 22-kHz USV bouts is shown for SERT^+/+^ (black bar), SERT^+/−^ (striped bar), and SERT^−/−^ (white bar) rats with sexes pooled (**A,C,E**) and separated by sex (**A’**,**C’**,**E’**), with females on the left and males on the right side of the panel. For rats that emitted bouts, number of calls per bout is also shown for rats with sexes pooled (**B,D,F**) and separated by sex (**B’,D’,F’**), with females on the left and males on the right side of the panel. *N* = 44 female (15 +/+, 15 +/− 14 −/−), *N* = 43 male (14 +/+, 15 +/−, 14 +/−) rats. Data are presented as mean ± SEM. G* *p* < 0.05 effect of genotype, with lines indicating significant post-hoc comparison between genotypes. S* *p* < 0.05 effect of sex. * *p* < 0.05 for subgroup comparison.

The temporal 22-kHz USV emission pattern during acquisition was also affected by genotype (G: F_2,87_ = 4.872, *p* = 0.010) and sex (S: F_1,87_ = 23.554, *p* < 0.001, GxS: F_2,87_ = 2.102, *p* = 0.129). The number of bouts emitted by SERT^−/−^ rats was lower than in SERT^+/−^ and SERT^+/+^ littermates (*p* = 0.044 and *p* = 0.004, respectively; Figure 4A). Similar to 22-kHz USV total calling time, the genotype effect was primarily seen in males due to the fact that female rats displayed less bouts than male rats in general (Figure 4A). When comparing bout length for vocalizing rats, however, no differences in the number of calls per bout were found between experimental conditions (G: F_2,87_ = 0.397, *p* = 0.676, Figure 3B; S: F_1,87_ = 0.757, *p* = 0.392, GxS: F_2,87_ = 0.088, *p* = 0.916 Figure 4B).

The temporal pattern of 22-kHz USV during extinction was not affected by genotype (G: F_2_ = 2.066, *p* = 0.133, Figure 4C) but sex (S: F_1,87_ = 8.777, *p* = 0.004, GxS: F_2,87_ = 2.066, *p* = 0.133, Figure 4C). Due to females not displaying any 22-kHz USV during extinction, their number of 22-kHz USV bouts emitted obviously differed from their male conspecifics. Bout length did not differ between experimental conditions (G: F_2,87_ = 2.199, *p* = 0.148, Figure 4D). 

During recovery, the effects on the temporal pattern of 22-kHz USV resemble the results from the extinction phase. Due to the nearly absent 22-kHz USV from female rats, sexes differed in the number of bouts emitted (S: F_1,87_ = 5.511, *p* = 0.021, Figure 4E’), with no effect of genotype (G: F_2,87_ = 2.526, *p* = 0.086, GxS: F_2,87_ = 1.355, *p* = 0.264, Figure 4E). Again, bout length did not differ between experimental conditions (G: F_2,87_ = 1.003, *p* = 0.338, Figure 3F; S: F_2,87_ = 0.226, *p* = 0.644, Figure 4F).

#### 2.2.4. CS+/CS- Presentation: Immobility

**Figure 5:**
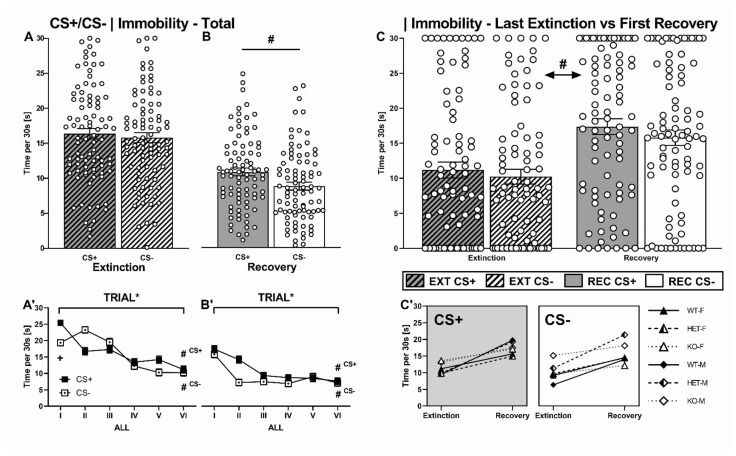
CS+/CS- presentation: immobility across all rats. Effects of SERT deficiency on immobility for CS+ and CS- presentations during extinction (**A**,**A’**) and recovery (**B**,**B’**), as well as the comparison of last trial extinction vs. first trial recovery (**C–C’**). Depicted are the total amounts of immobility for CS+ presentations (grey striped bar) and CS- presentations (white striped bar) during extinction (**A,C**), as well as CS+ presentation (grey bar) and CS- presentation (white bar) of recovery (**B,C**). Furthermore, single trial immobility levels for extinction (**A’**) and recovery (**B’**) are shown by means of CS+ presentations (black squares) and CS- presentations (white squares with dot). N = 87 rats. Data are presented as mean ± SEM. TRIAL* *p* < 0.05 effect of time course. # *p* < 0.05 significant within-subject comparison of various CS presentations. + *p* < 0.05 for within-subject comparison of first CS+ and CS- presentation. #CS+ *p* < 0.05 for within-subject comparison of first and last CS+ presentation. #CS- *p* < 0.05 for within-subject comparison of first and last CS- presentation.

**Figure 6:**
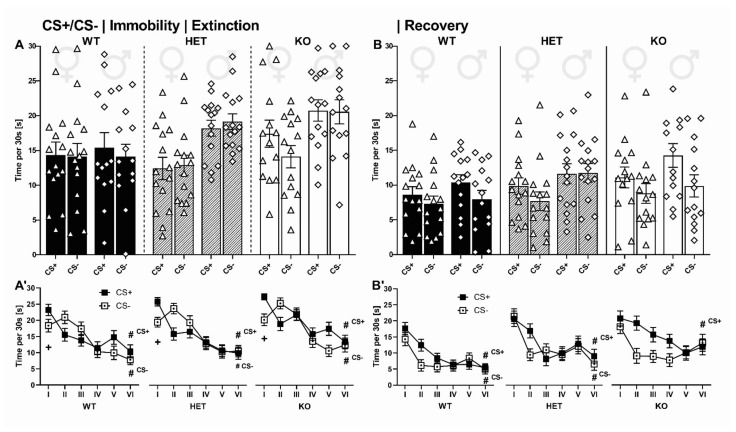
CS+/CS- presentation: Immobility, grouped by genotype and sex. Effects of SERT deficiency on immobility per 30 s time bin for CS+ and CS- presentation during extinction (**A**,**A’**) and recovery (**B**,**B’**). Depicted are the total amounts of immobility for both CS+ and CS- presentations for SERT^+/+^ (black bar), SERT^+/−^ (striped bar), and SERT^−^^/−^ (white bar) rats. Two bars on the left comprise CS+ and CS- presentations for females; males are shown on the two right bars of every genotype. Furthermore, single trial immobility levels for extinction (**A’**) and recovery (**B’**) are shown by means of CS+ presentations (black squares) and CS- presentations (white squares with dot). *N* = 29 SERT^+/+^ (15 female, 14 male), 30 SERT^+/−^ (15 female, 15 male), 28 SERT^−/−^ (14 female, 14 male) rats. Data are presented as mean ± SEM. TRIAL* *p* < 0.05 effect of time course. # *p* < 0.05 significant within-subject comparison of various CS presentations. + *p* < 0.05 for within-subject comparison of first CS+ and CS- presentation. #CS+ *p* < 0.05 for within-subject comparison of first and last CS+ presentation. #CS- *p* < 0.05 for within-subject comparison of first and last CS- presentation.

To determine the effects of differential fear conditioning, immobility levels displayed during CS+ and CS- presentations were compared separately for extinction and recovery. During extinction, there was no overall difference in immobility between CS+ and CS- presentations (CS: F_1.000,87_ = 1.643, *p* = 0.204, Figure 4A). Furthermore, immobility levels during CS+ and CS- presentations were not affected by genotype (CSxG: F_2.000,87_ = 2.331, *p* = 0.104) and sex (CSxS: F_1.000,87_ = 0.830, *p* = 0.365, Figure 5A). Throughout the trials of CS+ and CS- presentations, immobility decreased over the time course of extinction for both CS presentations (TRIAL: F_4.072,87_ = 49.414, *p* < 0.001). A comparison between the first and last CS presentation revealed a difference for either type of CS (CS+: T_86_ = 11.552, *p* < 0.001; CS-: T_86_ = 6.808, *p* < 0.001, Figure 5A’). As an indication of differential conditioning, immobility decreased more rapidly for CS+ than CS- presentations (TRIALxCS: F_4.689,87_ = 17.006, *p* < 0.001). Particularly high immobility levels were seen during the first CS+ presentation. This was not the case for the first CS- presentation, resulting in a prominent difference in immobility levels between the first CS+ and CS- presentation (T_86_ = 6.740, *p* < 0.001; Figure 5A’). The pattern of immobility during extinction remained unaltered despite SERT deficiency (TRIALxG: F_8.144,87_ =0.700, *p* = 0.694; TRIALxS: F_4.072,87_ =2.217, *p* = 0.066; Figure 6A). 

During recovery, immobility levels differed between CS+ and CS- presentations (CS: F_1.000,87_ = 12.653, *p* = 0.001, Figure 5B), regardless of genotype or sex (CSxG: F_2.000,87_ = 1.336, *p* = 0.269; CSxS: F_1.000,87_ = 0.053, *p* = 0.818). In general, CS+ presentations elicited more immobility than CS- presentations. Focusing on the course of immobility throughout successive trials of CS+ and CS- presentations, the response towards both CS+ and CS- presentations decreased over time (TRIAL: F_4.496,87_ = 25.046, *p* < 0.001), with CS- presentations showing a more rapid decline (TRIALxCS: F_4.262,87_ = 4.253, *p* = 0.002). Again, the amount of immobility elicited differed between the first and last trial for both stimuli (CS+: T_86_ = 7.174, *p* < 0.001; CS-: T_86_ = 6.165, *p* < 0.001, Figure 5B’). In accordance with extinction, the temporal pattern of declining immobility towards both CS+ and CS- presentations was seen across experimental conditions (TRIALxG: F_8.991,87_ =.994, *p* = 0.445; TRIALxS: F_4.496,87_ =.714, *p* = 0.599; Figure 6B’). 

Importantly, formerly extinguished behavior recovered. This is reflected in lower levels of immobility in response to the last CS presentation during extinction than in response to the first CS presentation during recovery a week later (EXT-REC: F_1.000,87_ = 26.622, *p* < 0.001, Figure 5C). Immobility during the last extinction trial was lower than during the first recovery trial for both CS+ and CS- presentations (CS: F_1.000,87_ = 2.502, *p* = 0.118, Figure 5C). The recovery effect was seen irrespective of genotype and sex (all *p* < 0.05, Figure 5C, for detailed depiction of experimental conditions, see Figure S1).

#### 2.2.5. CS+/CS- Presentation: 22-kHz USV

Similar to immobility, the overall emission of 22-kHz USV did not differ between CS+ and CS- presentations during extinction (CS: F_1.000,87_ = 0.005, *p* = 0.942, Figure S2A). Moreover, their emission was not modulated by genotype or sex (CSxG: F_2.000,87_ = 0.564, *p* = 0.571; CSxS: F_1.000,87_ = 0.005, *p* = 0.942, Figure S3A). However, the time spent calling 22-kHz USV varied throughout the time course of CS presentation (TRIAL: F_3.105,87_ = 2.778, *p* = 0.040, TRIALxCS: F_3.576,87_ = 1.120, *p* = 0.345 Figure S2A’). Similar to immobility, the 22-kHz USV emission differed between the first and last trial, yet only for CS+ but not CS- (CS+: T_86_ = 2.149, *p* = 0.034; CS-: T_86_ = 1.000, *p* = 0.320). This effect showed an interaction with sex (TRIALxS: F_3.105,87_ = 2.778, *p* = 0.040) but not genotype (TRIALxG: F_6.201,87_ = 1.330, *p* = 0.243). For male rats only—due to the absence of calling from their female conspecifics—22-kHz USV were emitted during early CS+ and CS- presentations, whereas no calling was detected during later time points.

In contrast to immobility, the emission of 22-kHz USV did not differ between CS+ and CS- presentations during recovery (CS: F_1.000,87_ = 3.493, *p* = 0.065, Figure S2B). As during extinction, their emission was not modulated by genotype or sex (CSxG: F_2.000,87_ = 1.007, *p* = 0.370; CSxS: F_1.000,87_ = 1.281, *p* = 0.261, Figure S3B). Again, throughout the time course of CS presentations, the emission of 22-kHz USV varied between sexes and genotypes – due to the virtual absence of calling from female rats and SERT^−/−^ rats during recovery (TRIAL: F_2.038,87_ = 6.475, *p* = 0.002; TRIALxS: F_3.105,87_ = 4.452, *p* = 0.013, TRIALxG: F_4.077,87_ = 2.531, *p* = 0.041, Figure S2B’). In fact, 22-kHz USV towards CS+ presentations showed a different time course than towards CS- presentations (TRIALxCS: F_2.305,87_ = 3.016, *p* = 0.044, Figure S2B’), with CS+ presentations showing higher levels during the first trials than CS- presentations, albeit the decrease did not reach statistical significance at the level of CS+ and CS- presentations (CS+: T_86_ = 0.752, *p* =0.454; CS-: T_86_ = -0.295, *p* = 0.7699). 

Importantly, no evidence for recovery of 22-kHz USV emission was evident (EXT-REC: F_1.000,87_ = 1.666, *p* = 0.685). 22-kHz emission for both CS+ and CS- presentations did not differ (CS: F_1.000,87_ = 1.348, *p* = 0.249).

### 2.3. Additional Behavioral Assays

With the aim to identify relevant factors associated with the effects of SERT deficiency on differential fear conditioning, including acquisition, extinction, and recovery, we tested rats in additional behavioral assays, namely activity box, elevated plus maze, novel object recognition, and hot plate. These assays further allowed us to identify trait-like inter-individual differences in novelty-seeking, anxiety-related behavior, habituation learning, cognitive performance, and pain sensitivity, and to test whether such inter-individual differences predict individual performance during differential fear conditioning.

#### 2.3.1. Activity Box

**Figure 7:**
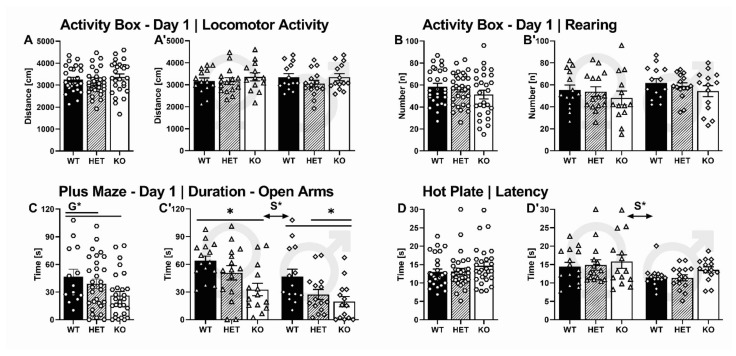
Additional behavioral assays. Effects of SERT deficiency on novelty-seeking in the activity box (**A**–**B’**), anxiety-like behavior in the elevated plus maze (**C**,**C’**), and pain sensitivity in the hot plate test (**D**,**D’**). Depicted are measures for SERT^+/+^ (black bar), SERT^+/−^ (striped bar), and SERT^−/−^ (white bar) rats with sexes pooled (**A**–**D**) and separated by sex (**A’,B’**,**C’,D’**), with females on the left and males on the right side of the panel. Measures are (**A**) distance travelled in the activity box; (**B**) number of rearings in the activity box; (**C**) time spent in open arms of the elevated plus maze; and (**D**) latency to withdraw a paw in the hot plate test. N = 44 female (15 +/+, 15 +/−, 14 −/−), N = 43 male (14 +/+, 15 +/−, 14 −/−) rats, except activity box (see materials and methods for details). Data are presented as mean ± SEM. G* *p* < 0.05 effect of genotype, with lines indicating significant post-hoc comparison between genotypes. S* *p* < 0.05 effect of sex. * *p* < 0.05 for subgroup comparison.

First, rats were tested in a small open field on two consecutive days to screen for novelty-seeking and habituation learning. SERT deficiency had only minor effects on horizontal and vertical locomotor activity. On both days, no differences in distance travelled were found between genotypes (G_DAY1_: F_2,85_ = 1.202, *p* = 0.306; G_DAY2_: F_2,84_ = 1.632, *p* = 0.202; Figure 7A) and sexes (S_DAY1_: F_1,85_ = 0.051, *p* = 0.822; S_DAY2_: F_1,84_ = 0.743, *p* = 0.391; GxS_DAY1_: F_2,85_ = 0.373, *p* = 0.690; GxS_DAY2_: F_2,84_ = 1.244, *p* = 0.294, Figure 7A’). Moreover, on day 1, no differences in rearing behavior were present (G_DAY1_: F_2,85_ = 1.311, *p* = 0.275, Figure 7B; S_DAY1_: F_1,85_ = 2.538, *p* = 0.115; GxS_DAY1_: F_2,85_ = 0.015, *p* = 0.985, Figure 7B’). On day 2, vertical activity differed between genotypes (G_DAY2_: F_2,84_ = 9.263, *p* < 0.001), with SERT^−/−^ rats displaying less rearing behavior than SERT^+/−^ and SERT^+/+^ littermates (*p* = 0.001 and *p* < 0.001, respectively). Rearing behavior was not influenced by sex (S_DAY2_: F_1,84_ = 0.060, *p* = 0.807; GxS_DAY2_: F_2,84_ = 326, *p* = 0.723). Across days, distance travelled declined, reflecting habituation learning (DAY: F_2.000,82_ = 99.501, *p* < 0.001). This decline was modulated by genotype (DAYxG: F_2.000,82_ = 4.904, *p* = 0.010) but not sex (DAYxS: F_1.000,82_ = 0.487, *p* = 0.488, DAYxGxS: F_2.000,82_ = 0.277, *p* = 0.759), with the most rapid decline in distance travelled displayed by SERT^−/−^ rats compared to their SERT^+/−^ and SERT^+/+^ littermates (*p* = 0.007 and *p* = 0.016, respectively). Likewise, rearing behavior declined across days (DAY: F_1.000,82_ = 115.170, *p* < 0.001), but this decline was not modulated by genotype (DAYxG: F_2.000,82_ = 2.335, *p* = 0.104) or sex (DAYxS: F_1.000,82_ = 2.735, *p* = 0.102, DAYxGxS: F_2.000,82_ = 0.287, *p* = 0.752). 

#### 2.3.2. Elevated Plus Maze

Next, rats were screened for anxiety-like behavior in the elevated plus maze on two consecutive days. SERT deficiency was associated with enhanced anxiety-related behavior. On the first day, open arm time differed between genotypes (G_DAY1_: F_2,87_ = 9.917, *p* < 0.001, Figure 7C). SERT^+/+^ rats spent more time in open arms than SERT^+/−^ and SERT^−/−^ littermates (*p* = 0.012 and *p* < 0.001, respectively), indicating that SERT deficiency leads to enhanced levels of anxiety-like behavior as reflected by avoidance of open spaces. Although female rats spent more time in open arms than their male conspecifics (S_DAY1_: F_1,87_ = 11.405, *p* = 0.001, GxS_DAY1_: F_2,87_ = 0.325, *p* = 0.724, Figure 7C’), the anxiogenic effect of SERT deficiency was robust and SERT^−/−^ rats of both sexes displayed more anxiety-related behavior than their SERT^+/+^ littermates (*p* = 0.002 and *p* = 0.005, respectively). On the second day, female rats still spent more time in open arms than their male conspecifics (S_DAY2_: F_1,87_ = 5.366, *p* = 0.023), yet no genotype differences were detected (G_DAY2_: F_2,87_ = 2.266, *p* = 0.110, GxS_DAY2_: F_2,87_ = 0.156, *p* = 0.856), partly due to the reduction in open arm time displayed by all experimental conditions irrespective of genotype and sex, reflecting intact contextual memory and the ability to adjust exploratory behavior in an anxiogenic environment (DAY: F_1.000,87_ = 14.510, *p* < 0.001; DAYxG: F_2.000,87_ = 1.589, *p* = 0.210; DAYxS: F_1.000,87_ = 0.104, *p* = 0.709, DAYxGxS: F_2.000,87_ = 0.260, *p* = 0.771). Overall locomotor activity in the elevated plus-maze on the first day was not affected by genotype (G_DAY1_: F_2,87_ = 2.834, *p* = 0.065) but sex, with females displaying higher levels of locomotor activity than males (S_DAY1_: F_2,87_ = 6.878, *p* = 0.010; GxS_DAY1_: F_2,87_ = 1.291, *p* = 0.281). On the second day, no differences between genotypes and sexes were apparent (G_DAY2_: F_2,87_ = 0.467, *p* = 0.629; S_DAY2_: F_2,87_ = 2.679, *p* = 0.106; GxS_DAY2_: F_2,87_ = 0.453, *p* = 0.638). Locomotor activity declined across days (DAY: F_1.000,87_ = 77.413, *p* < 0.001), irrespective of genotype (DAYxG: F_2.000,87_ = 0.665, *p* = 0.417) and sex (DAYxS: F_1.000,87_ = 0.806, *p* = 0.450, DAYxGxS: F_2.000,87_ = 0.312, *p* = 0.733).

#### 2.3.3. Novel Object Recognition 

Cognitive performance in the novel object recognition test was not affected by SERT deficiency. The ability to recognize familiar objects and to differentiate them from novel objects was tested in the novel object recognition test after a delay of 30 min. When given the opportunity to explore a novel object simultaneously with a familiar object, rats preferred the novel object independent of genotype and sex (OBJ: F_1.000,87_ = 54.416, *p* < 0.001, OBJxG: F_2.000,87_ =0.257, *p* = 0.774, OBJxS: F_1.000,87_ = 1.183, *p* = 0.280, OBJxGxS: F_1.000,87_ = 0.086, *p* = 0.918, Figure S4), as reflected in novel object investigation times above chance level in all experimental conditions (all *p* < 0.05). Of note, object exploration displayed during the acquisition phase did not differ between genotypes (G: F_2.000,87_ = 1.960, *p* = 0.148) but sex, with males exploring more than females (S: F_1.000,87_ = 19.724, *p* < 0.001, GxS: F_2.000,87_ = 0.249, *p* = 0.781). 

#### 2.3.4. Hot Plate

Effects of SERT deficiency on pain reactivity to thermal stimulation were not found. The latencies to withdraw the paws from a hot surface were comparable between genotypes (G: F_2,87_ = 1.200, *p* = 0.307, Figure 7D). However, female rats displayed higher withdrawal latencies compared to their male conspecifics (S: F_1,87_ = 8.488, *p* = 0.005, GxS: F_2,87_ = 0.131, *p* = 0.877, Figure 7D’), indicating less pain reactivity to thermal stimulation in females.

### 2.4. Predictors of Inter-Individual Differences in Immobility

**Figure 8:**
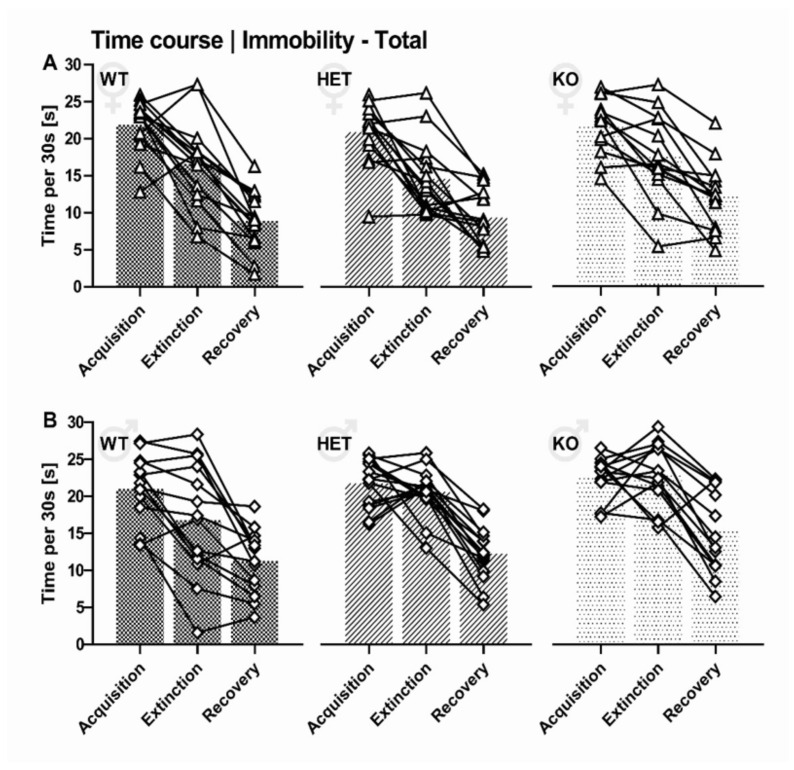
Inter-individual differences in immobility. Effects of SERT deficiency on immobility during acquisition, extinction, and recovery, depicted as individual values for female (**A**, with triangles) and male (**B**, white squares) rats. Additionally, means for SERT^+/+^ (checkered bars), SERT^+/−^ (striped bars), and SERT^−/−^ (dotted bars) rats are shown. N = 44 female (15 +/+, 15 +/+, 14 −/−), N = 43 male rats (14 +/+, 15 +/+, 14 −/−) rats.

**Figure 9:**
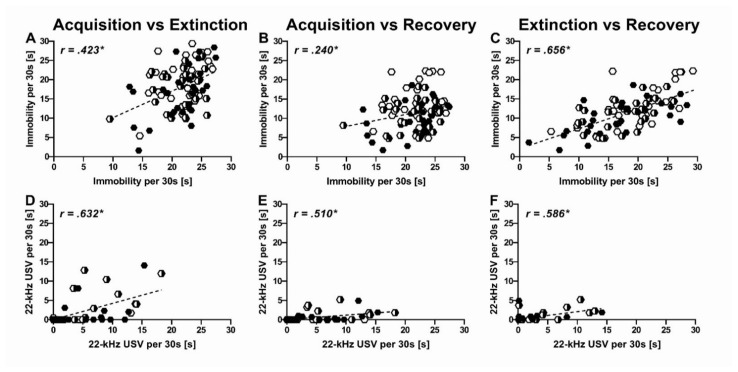
Correlation between immobility and 22-kHz USV emission. Duration of immobility (**A–C**) and 22-kHz USV (**D–F**) during acquisition in relation to extinction (**A,D**) and recovery (**B,E**), and during extinction and recovery (**C,F**), respectively, with individual values for SERT^+/+^ (black diamond), SERT^+/−^ (black-white diamond), and SERT^−/−^ (white diamond) rats. *N* = 29 SERT^+/+^ (15 female, 14 male), 30 SERT^+/−^ (15 female, 15 male), 28 SERT^−/−^ (14 female, 14 male) rats. Statistical significance (*p* < 0.05) of correlation coefficients is indicated in bold and italic.

Overall, rats spending a lot of time immobile during the acquisition phase were also prone to show higher levels of immobility during subsequent phases of the differential fear conditioning paradigm, namely extinction and recovery (Figure 8). For one, immobility displayed in response to tone-shock pairings during acquisition (ACQ) was positively associated with immobility during extinction (ACQ-EXT: r = 0.423, *p* < 0.001, Figure 9A) and recovery (ACQ-REC: r = 0.240, *p* = 0.025, Figure 9B). Even more so, immobility levels in the extinction context reliably predicted immobility levels during recovery in the same context seven days later (EXT-REC: r = 0.656, *p* < 0.001, Figure 9C). This indicates that variability in immobility was in part due to stable inter-individual differences that were reliably identified throughout the different phases of the differential fear conditioning paradigm.

Similar to immobility, there were stable inter-individual differences in the emission of 22-kHz USV. Specifically, 22-kHz USV emission during acquisition was positively associated with 22-kHz USV emission during extinction (ACQ-EXT: r = 0.632, *p* < 0.001, Figure 9D) and recovery (ACQ-REC: r = 0.510, *p* < 0.001, Figure 9E). Moreover, 22-kHz USV emission during extinction was positively associated with 22-kHz USV emission during recovery (EXT-REC: r = 0.586, *p* < 0.001, Figure 9F). Unexpectedly, however, the association between 22-kHz USV emission and immobility was weak. While there was a positive correlation during acquisition (ACQ-ACQ: r = 0.259, *p* < 0.015, Figure S5A), this was not the case during extinction (EXT-EXT: r = 0.130, *p* = 0.230, Figure S5B) and recovery (REC-REC: r = 0.087, *p* = 0.425, Figure S5C). Moreover, 22-kHz USV emission in response to tone-shock pairings during acquisition did not predict immobility levels during extinction (ACQ-EXT: r = 0.116, *p* = 0.284) and recovery (ACQ-REC: r = 0.028, *p* = 0.800). This suggests that 22-kHz USV emission and immobility are at least partially distinct components of the fear response. Of note, body weight was correlated with inter-individual differences in immobility in females during extinction but not acquisition or recovery (BW-ACQ: r = 0.055, *p* = 0.724; AB-EXT: r = 0.405, *p* = 0.007; AB-REC: r = 0.231, *p* = 0.136). No prominent association between body weight and immobility was obtained in males (BW-ACQ: r = −0.091, *p* = 0.566; AB-EXT: r = −0.156, *p* = 0.324; AB-REC: r = −0.041, *p* = 0.797). Body weight did not correlate with the emission of 22-kHz USV in males and females (all *p* > 0.05).

**Figure 10:**
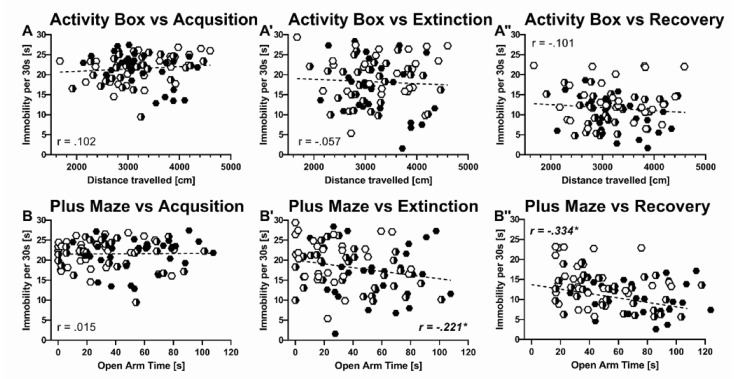
Correlation: novelty-seeking, anxiety-related behavior, and immobility. Duration of immobility during acquisition, extinction, and recovery, in relation to novelty-seeking in the activity box (**A**–**A’’**) and anxiety-like behavior in the elevated plus maze (**B**–**B’’**). Depicted are SERT^+/+^ (black diamond), SERT^+/−^ (black-white diamond), and SERT^−/−^ (white diamond) rats. *N* = 29 SERT^+/+^ (15 female, 14 male), 30 SERT^+/−^ (15 female, 15 male), 28 SERT^−/−^ (14 female, 14 male) rats, except activity box (see materials and methods for details). Data are presented as individual values and correlation coefficients. Statistical significance (*p* < 0.05) of correlation coefficient is indicated in bold and italic.

Because recovery was reflected in immobility levels but not 22-kHz USV emission, we thus asked whether immobility during differential fear conditioning can be predicted by trait-like inter-individual differences in novelty seeking, anxiety-related behavior, habituation learning, cognitive performance, and pain sensitivity. Locomotor activity during the first exposure to the activity box (AB) did not predict immobility levels displayed during acquisition, extinction, or recovery (AB-ACQ: r = 0.102, *p* = 0.355; AB-EXT: r = −0.057, *p* = 0.603; AB-REC: r = −0.101, *p* = 0.358, Figure 10A-A’’). Likewise, habituation learning displayed in response to the repeated exposure to the activity box did not predict immobility levels (AB-ACQ: r = −0.093, *p* = 0.405; AB-EXT: r = −0.118, *p* = 0.287; AB-REC: r = −0.115, *p* = 0.302. Avoidance of the open arms in the elevated plus maze (EPM), however, was associated with higher levels of immobility during extinction and recovery (EPM-EXT: r = 0.423, *p* < 0.001, Figure 10B’; EPM-REC: r = 0.240, *p* = 0.025, Figure 10B’’) but not in the acutely threating situation of tone-shock pairings during acquisition (EPM-ACQ: r = 0.015, *p* = 0.893, Figure 10B). The typically seen reduction in open arm time from the first to the second exposure to the elevated plus maze was not associated with immobility levels displayed during acquisition, extinction, or recovery (EPM-ACQ: r = 0.075, *p* = 0.490; EPM-EXT: r = 0.016, *p* = 0.883; EPM-REC: r = 0.065, *p* = 0.551). Finally, immobility during different phases of differential fear conditioning was neither predicted by cognitive performance in the novel object recognition test (OBJ-ACQ: r = −0.086, *p* = 0.431; OBJ-EXT: r = 0.016, *p* = 0.882; OBJ-REC: r = 0.017, *p* = 0.877) nor pain sensitivity in the hot plat test (HP-ACQ: r = 0.059, *p* = 0.589; HP-EXT: r = −0.075, *p* = 0.489; HP-REC: r = −0.038, *p* = 0.725).

## 3. Discussion

In the present study, we aimed at identifying key factors associated with inter-individual differences in fear extinction in rats and assessed fear-related behavior through quantifying the emission of alarm 22-kHz USV in addition to the commonly applied measure immobility. We found that SERT deficiency strongly affected the emission of 22-kHz USV during differential fear conditioning. During acquisition, extinction, and recovery, SERT deficiency consistently led to a reduction in 22-kHz USV emission. In line with our previous report [37], most 22-kHz USV were emitted during acquisition when repeated tone-shock pairings were presented, whereas calling behavior declined in subsequent phases or was abolished completely depending on genotype and sex. Specifically, besides their already reduced 22-kHz USV emission rates during acquisition, rats lacking SERT but not wildtype littermate controls virtually ceased to emit 22-kHz USV during extinction and subsequent recovery. This was seen in male and female rats, albeit 22-kHz USV emission was comparatively low in females. A different pattern was evident for immobility. SERT deficiency did not affect immobility during acquisition. Genotype differences started to emerge during extinction, however, and during recovery rats lacking SERT showed much higher levels of immobility than wildtype littermate controls. With the aim to identify relevant factors associated with the effects of SERT deficiency on differential fear conditioning, we tested rats in additional behavioral assays, namely activity box, elevated plus maze, novel object recognition, and hot plate. Rats lacking SERT behaved similar to their littermates in those assays, with the expectation of the elevated plus maze, where they engaged in considerably higher levels of anxiety-related behavior. Finally, we studied predictors of inter-individual differences in immobility during differential fear conditioning and found that immobility displayed in response to tone-shock pairings during acquisition predicted immobility levels during extinction and recovery. The predictive quality of 22-kHz USV was low. Among the trait-like inter-individual differences in novelty seeking, anxiety-related behavior, habituation learning, cognitive performance, and pain sensitivity, anxiety-related behavior had the best predictive quality.

### 3.1. Fear-Related Behavior

Tone-shock pairings during the acquisition phase of the differential fear conditioning paradigm led to high levels of immobility, followed by a gradual decline throughout phases of extinction and recovery. During extinction, immobility towards the tone that was previously accompanied by an electric stimulation (CS+) was stronger at the outset and decreased more rapidly in the course of training, compared to the stimulus that was never paired with foot shocks (CS-). Similarly, during recovery, rats discriminated between CS+ and CS- presentations and displayed higher immobility levels during CS+ presentations. For both CS presentations, extinguished behavior recovered, as reflected in the lower levels of immobility in response to the last CS presentation during extinction than in response to the first CS presentation during recovery a week later. Thus, our results indicate that differential conditioning was accompanied by substantial conditioning towards the CS- that was never paired with foot shocks. Various possibilities for substantial conditioning towards the CS- are conceivable. Due to similarities between CS+ and CS-, fear responses induced by the CS+ might have been generalized to the CS- [56]. Moreover, the context might have contributed to the responding towards the CS-. Fear-related responses can be induced by the context itself and a clear discrimination of context and cue in fear conditioning paradigms is difficult, where the context can be interpreted as combination of different cues [56]. Even though contextual cues, most notably odor and visual patterning, differed between acquisition and extinction, there might have been some degree of generalization between the contexts, which may have elevated responding towards the CS-.

Consistent with our previous studies, the majority of 22-kHz USV was emitted during acquisition [37,52,53,54,55,56,57,58,59]. Much lower 22-USV emission rates were seen during extinction and recovery. No recovery effect was evident for 22-kHz USV emission. In contrast to immobility, the emission of 22-kHz USV did not differ between CS+ and CS- presentations during extinction, whereas during recovery, there was evidence for differential 22-kHz USV emission towards CS+ and CS-.

#### 3.1.1. Sex Differences in Fear-Related Behavior

Male and female rats showed equal levels of immobility during acquisition. During extinction and recovery, however, female rats displayed lower levels of immobility. Consistent sex differences were seen in the emission of 22-kHz USV during acquisition, extinction, and recovery of differential fear conditioning. During acquisition, female rats showed a lower prevalence of 16% to emit 22-kHz USV compared to their male conspecifics with 63%, similar to our previous report [37]. During extinction and recovery, female rats virtually ceased to emit 22-kHz USV with the exception of one vocalizing female during recovery as compared to 40% of males during extinction and still 30% during recovery. The low prevalence of calling in female rats is reflected in less time spent calling and fewer numbers of bouts emitted as well. Interestingly, vocalizing females showed no difference in the temporal 22-kHz USV emission pattern and bout length did not differ between males and females in contrast to previous findings [37]. This inconsistency might be due to the fact that a differential fear conditioning paradigm was applied here. Even though the number of tone-shock pairings was the same in the present and the previous study, the addition of CS- presentations might have affected the temporal 22-kHz USV emission pattern. For instance, the additional tone presentations could have interfered with emitting 22-kHz USV in bouts. 

The mechanism underlying sex differences in 22-kHz USV emission remains unclear. For one, similar sex differences were seen in other aversive experimental settings, with female rats emitting fewer 22-kHz USV when confronted with air puffs [45,46] or electric shocks [60,61,62]. Interestingly, there is a report on reduced 22-kHz USV emission in female rats despite higher foot shock sensitivity, where relatively low shock levels were found to be sufficient to induce sonic squeaks associated with pain in female but not male rats [63]. Therefore, dose-response curves for 22-kHz USV induced by foot shock application might differ between male and female rats. For male rats, a positive relation between foot shock intensity and 22-kHz USV was reported, with foot shocks of 0.5 mA being sufficient to induce 22-kHz USV in the majority of rats [57]. For female rats, dose-response studies on 22-kHz USV evoked by foot shocks are still to be conducted and it appears possible that the higher sensitivity to foot shocks in females might result in greater perceived imminence of threat, which in turn can lead to the immediate cessation of 22-kHz USV emission [64]. In the present study, however, female rats displayed lower pain sensitivity in the hot plate test performed shortly after the differential fear conditioning, complicating the relation of 22-kHz USV, sensitivity to foot shock, and perception of pain.

On the other hand, opposing sex differences in 22-kHz USV emission were seen under more naturalistic conditions. In response to a predator, female rats living in social groups in a visible burrow system emit more 22-kHz USV than their male conspecifics [65,66]. Other than solely expressing anxiety and fear, 22-kHz USV are thought to function as alarm calls to warn conspecifics about threats and were shown to evoke a fear response in receiver rats [42,65]. The latter was also observed during social fear conditioning [59,67,68] and confirmed in playback studies, where 22-kHz USV induced behavioral inhibition in receiver rats [43,69]. Supporting a communicative function, 22-kHz USV emission was reported to be potentiated by the presence of conspecifics [42] and it appears possible that this audience effect is more prominent in female than male rats.

Finally, the direction of sex differences in 22-kHz USV evoked by foot shock may be heavily influenced by strain. Male Sprague-Dawley rats were reported to produce more 22-kHz USV than their female conspecifics, whereas the opposite was shown for Long-Evans rats [61]. Interestingly, the studies conducted in naturalistic environments used Long-Evans rats, whereas sex differences in 22-kHz USV induced by foot shock were obtained in Sprague-Dawley rats [63], and 22-kHz USV elicited by air puffs in outbred Wistar and inbred F344 rats [46]. A systematic study on sex differences in the emission of 22-kHz USV including multiple elicitors of 22-kHz USV appears therefore to be warranted.

#### 3.1.2. Genotype Differences in Fear-Related Behavior

Effects of SERT deficiency did not affect immobility during the acquisition phase of the differential fear conditioning paradigm and no differences were seen between SERT^+/+^, SERT^+/−^, and SERT^−/−^ rats. This is in line with previous studies and suggests intact acquisition of conditioned fear despite SERT deficiency [30,32,33,37]. During extinction on the next day, however, effects of SERT deficiency started to emerge and prominent genotype differences were evident during the recovery phase a week later. Here, SERT^−/−^ rats, especially SERT^−/−^ males, displayed higher levels of immobility compared to SERT^+/+^ and SERT^+/−^ rats. This supports previous findings obtained in rats lacking SERT [28], which displayed a reduced ability to extinguish fear-related behavior in several studies [23,25,29,30,31,32,33,34,35], with some of them suggesting that the lack of SERT also impedes extinction recall [32,34]. In our own previous study, however, we did not see an effect of SERT deficiency on extinction [37]. The fact that genotype differences were evident in the present study might be related to the presentation of a CS- in addition to a CS+. The CS- presentation might have helped to reveal genotype effects due to a weakening of ceiling effects of otherwise high baseline immobility levels elicited by tone-shock pairings.

Similar to sex, SERT deficiency also strongly affected the emission of 22-kHz USV during differential fear conditioning. This is consistent with our previous report [37]. During acquisition, only 18% of SERT^−/−^ rats emitted 22-kHz USV compared to 52% of SERT^+/+^ and 47% of SERT^+/−^ rats. Similar patterns were obtained during extinction and recovery, although at a much lower level. In line with the significantly lower prevalence in 22-kHz USV of SERT^−/−^ rats, the total calling time of SERT deficient rats was significantly reduced in all phases of differential fear conditioning. Additionally, SERT^−/−^ male rats emitted less bouts during acquisition. Yet again, bout length did not differ between genotypes.

There are many neurobiological differences potentially contributing to these genotype effects. One of them is alterations in the function of the amygdala. The amygdala is known to play a key role in the acquisition of fear [70] and the production of 22-kHz USV was found to be orchestrated by an interplay of several nuclei of the amygdala [71]. For example, the medial nucleus [72] as well as the basolateral nucleus of the amygdala [73] were reported to mediate 22-kHz USV during conditioned avoidance behavior. However, the central nucleus of the amygdala is widely believed to exert the most dramatic effects on 22-kHz USV production [71]. In fact, removal [74] or neurotoxic lesion [75] of the central nucleus of the amygdala blocked 22-kHz USV as a fear-related conditional response. Interestingly, SERT^−/−^ rats exhibit diverging neuronal activity in the central nucleus of the amygdala [33] and this might contribute to changes in 22-kHz USV production. Other factors potentially contributing to the genotype differences in fear-related behavior are alterations in 5-HT receptor expression or sensitivity. In fact, SERT deficiency was reported to be associated with changes in 5-HT receptors. In SERT^−/−^ rats, the 5-HT1A receptor is desensitized [76,77,78] and 5-HT3 receptor function is altered [79]. In SERT^−/−^ mice, 5-HT1A and 5-HT1B autoreceptor binding and function is altered [80,81], as well as 5-HT2A/2C receptor density [82,83]. These changes are observed in a region-specific manner. Such receptor changes could have contributed to the changes in immobility and 22-kHz USV emission. Future studies employing (local) genetic or pharmacological manipulations will be needed for further understanding.

### 3.2. Relation of 22-kHz USV and Immobility

While sex differences in immobility and 22-kHz USV emission point in the same direction, with male rats spending more time immobile and vocalizing more than female rats, genotype differences are inconsistent. Despite the fact that rats lacking SERT display more immobility, they do not emit more 22-kHz USV than littermate controls. A dissociation of immobility and 22-kHz USV emission has been previously reported [37,45]. Although immobility and 22-kHz USV emission are both thought to reflect enhanced anxiety and fear, this suggests that they reflect at least in part different aspects of the fear response. In rats lacking SERT, high levels of immobility were found to be associated with exaggerated threat-related bradycardia and related findings were obtained in human carriers of the short 5-HTTLPR allelic variant [35]. Because heart rate was linked to 22-kHz USV emission, this could explain why 22-kHz USV emission is reduced in rats lacking SERT. In fact, pharmacological studies targeting β-adrenergic receptors suggest that the emission of 22-kHz USV is positively associated with heart rate [84]. Moreover, acoustic features, such as small changes in peak frequency, were reported to be correlated with blood pressure and heart rate [85]. 

The view that immobility and 22-kHz USV emission reflect at least in part different aspects of the fear response is supported by the fact that they were positively correlated during the acquisition phase but not during extinction and recovery. Moreover, 22-kHz USV emission during tone-shock pairings did not predict immobility levels during subsequent phases, suggesting a low predictive quality of the acute 22-kHz USV response for conditioned fear. This dissociation might be due to only partially overlapping neuronal circuits controlling both components of the fear response. In fact, diverging pathways from the basolateral nucleus of the amygdala and the central nucleus of the amygdala were associated with immobility and 22-kHz USV emission, respectively. It was shown that neurotoxic lesions of the basolateral nucleus of the amygdala impair both immobility and 22-kHz USV, whereas neurotoxic lesions of the central nucleus of the amygdala impair the emission of 22-kHz USV in a greater fashion [75].

Together, this indicates that the emission of 22-kHz USV is associated with a negative affective state but that their emission is not a simple reflection of a negative affective state. A sufficiently strong negative affective state appears to be a necessary condition for 22-kHz USV to occur. However, whether a negative affective state indeed leads to 22-kHz USV appears to be dependent on other factors, such as sex, genotype, and social factors, including the presence of conspecifics [42]. This is most likely due to the communicative function of 22-kHz USV as alarm calls [42,65]. Immobility, in contrast, has different functions and is considered to occur in an attentive action preparation phase during threat exposure. While 22-kHz USV emission most likely increases the risk of being detected by a predator, immobility is supposed to reduce the likelihood of being detected. A careful differentiation between affective state and the expression of the affective state appears warranted.

### 3.3. Trait-Like Inter-Individual Differences

Sexes did not differ in novelty-seeking and habituation learning in the activity box and cognitive performance during novel object recognition was similar in male and female rats. In the elevated plus maze, however, male rats displayed more anxiety-related behavior than female rats. Moreover, pain sensitivity was higher in male than female rats. Both sex differences might be associated with the differences between male and female rats seen during differential fear conditioning. Heightened anxiety and pain sensitivity in male rats might have contributed to the higher levels of immobility during extinction and recovery and the increased level of 22-kHz USV emission throughout all three phases of differential fear conditioning, albeit elevated levels of 22-kHz USV in male rats was evident in our previous report in absence of differences in pain sensitivity [37].

SERT deficiency had no major effects on novelty-seeking in the activity box, cognitive performance assessed during novel object recognition, and pain sensitivity quantified in the hot plate test. This is consistent with previous reports [22,37,86]. Of note, novel recognition deficits were previously reported in studies applying longer inter-trial intervals of more than 30 min [86,87]. Therefore, genotype differences in 22-kHz USV emission and immobility displayed during differential fear conditioning do not appear to be due to unspecific effects associated with altered levels of exploratory behavior, such as very low or very high activity levels. Moreover, severe cognitive impairments can be excluded as the driving force. Likewise, differences in pain sensitivity do not appear to play a major role. Finally, the fear extinction deficit displayed by rats lacking SERT does not appear to be due to a general deficit in habituation learning. In fact, rats lacking SERT displayed the most robust decline in locomotor activity when exposed to the activity box a second time.

However, SERT deficiency affected anxiety-related behavior in the elevated plus maze. In both sexes, a clear gene dosage effect was evident and anxiety-related behavior was robustly increased in rats lacking SERT. Increased anxiety-related behavior was consistently seen in previous studies applying the elevated plus maze [22,23,24,25,26,88] as well as other paradigms suitable to reveal effects on anxiety, such as light-dark test [22,23,24,25,88] and novelty suppressed feeding [25]. This suggests that the alterations displayed by rats lacking SERT during differential fear conditioning might at least partly be driven by higher trait anxiety. 

However, this does not appear to be the case for 22-kHz USV emission. The increase in anxiety-related behavior appears to be in contrast to the reduction in 22-kHz USV emission displayed by rats lacking SERT. In previous studies, 22-kHz USV emission was linked to high trait anxiety, and it was shown that rats that displayed high levels of anxiety-related behavior in the elevated plus maze emitted particularly high numbers of 22-kHz USV when challenged with tone-shock pairings during fear conditioning [53]. Therefore, one would have expected higher levels of 22-kHz USV emission in rats lacking SERT and not lower 22-kHz USV emission rates. In contrast, the increase in anxiety-related behavior displayed by rats lacking SERT might underlie the higher level of immobility during the recovery phase of the differential fear conditioning paradigm. In fact, trait anxiety was found to predict enhanced fear memory after fear conditioning in mice [89] and rats [68]. Moreover, rats selectively bred for high anxiety display deficits in extinction and extinction recall [90]. Related to that, inter-individual differences in extinction were found to result in systematic variation in recovery, where slow extinguishing rats were more prone to the relapse of fear than their fast-extinguishing conspecifics [91]. Finally, there is also evidence that anxious rats display higher levels of immobility [53,92], enhanced discrimination of fear-relevant cues [93], but slower active avoidance learning [94] associated with alterations in 5-HT concentrations [95].

Finally, SERT deficiency also affected body weight gain and body weight was reduced in rats lacking SERT. This effect was most prominent in males. Reports on the effects of SERT deficiency on body weight are rare, with a few exceptions. For example, one study found a reduction only during early life [87] and another one only in females [21] but not in males, as in the present study. However, it appears unlikely that body weight had prominent effects. Correlations between body weight and immobility or 22-kHz USV emission were rarely observed. While it is difficult to see how the genotype difference in body weight might directly contribute to the behavioral differences, body weight is often used as a proxy for the rank in a social hierarchy and the dominance structure was shown to be associated with the emission of 22-kHz USV [96]. Moreover, it was reported that social dominance status in rats predicts social fear transmission in rats. Following a social interaction with a fear conditioned dominant rat, subordinate rats displayed enhanced fear responses, possibly driven by the emission of 22-kHz USV [97]. It thus would be interesting to test whether SERT deficiency affects the social hierarchy. 

### 3.4. Clinical Implications

In humans, the 5-HT system including the 5-HTTLPR plays a key role in the etiology of anxiety disorders and affects treatment efficacy. Specifically, the short allelic variant associated with reduced transcription and altered function of SERT leads to an increased risk of developing PTSD after high trauma exposure [19] and reduces treatment efficacy of exposure-based therapy [98]. While immediate results of exposure-based therapy were found to be indistinguishable in long and short allele carriers [99], short but not long allele carriers displayed strong return of fear [98,99], similar to SERT deficient rats in the present study. Because amygdala activation was repeatedly associated with differences in fear extinction across species [18,25,33,100], it would be interesting to see whether targeted amygdala manipulations might help to improve fear extinction in SERT deficient rats.

## 4. Conclusions

Our results show that SERT deficiency strongly affected the emission of 22-kHz USV during differential fear conditioning. During acquisition, extinction, and recovery, SERT deficiency consistently led to a reduction in 22-kHz USV emission. While SERT deficiency did not affect immobility during acquisition, genotype differences started to emerge during extinction, and during recovery rats lacking SERT showed higher levels of immobility than wildtype littermate controls. Recovery was reflected in increased levels of immobility but not 22-kHz USV emission. Prominent sex differences were evident. Among several measures for trait-like inter-individual differences, anxiety-related behavior had the best predictive quality.

## 5. Materials and Methods

### 5.1. Animals and Housing

The effects of SERT deficiency on extinction and recovery in a differential fear conditioning paradigm were tested in male and female constitutive homozygous SERT^−/−^ and heterozygous SERT^+/−^ mutant rats, as compared to their wildtype SERT^+/+^ littermate controls. SERT^−/−^ rats completely lacking 5-HTT (SLC6A41Hubr) were generated by N-ethyl-N-nitrosurea (ENU) [101] and outcrossed with commercially available Wistar rats (Harlan, Ter Horst, The Netherlands) for at least 10 generations [21]. In total, *N* = 87 rats were included (*N* = 43 female rats (14 +/+, 15 +/−, 14 −/−), *N* = 44 male rats (15 +/+, 15 +/−, 14 −/−)). Rats were identified by paw tattoo and genotyping was performed as previously described [37].

To obtain SERT^−/−^ and SERT^+/−^ offspring together with SERT^+/+^ littermate controls, a heterozygous breeding strategy was applied as before [37]. Briefly, female and male SERT^+/−^ rats were paired for breeding. To avoid genetic drifts, male and female SERT^+/−^ breeders were obtained by outcrossing SERT^+/−^ males with Wistar females (Harlan, Ter Horst, The Netherlands). In order to avoid litter effects, only litters with all three genotypes were included in the experiments. After weaning on postnatal day 21, rats were socially housed in mixed-genotype groups of *N* = 4–5 with same-sex littermate partners in standard Macrolon Type IV cages with high stainless-steel covers (58 × 33 × 20 cm) and bedding in an animal room with a 12:12 h light-dark cycle (lights on from 7 a.m. to 7 p.m.). Standard rodent chow (Altromin, Lage, Germany) and water (0.0004% HCl solution) were available ad libitum.

### 5.2. General Procedure

Rats were tested with 2–4 months of age. After a standardized handling procedure on three consecutive days, the following behavioral assays were performed in the following order: activity box, elevated plus maze, novel object recognition, differential fear conditioning, and hot plate. The interval between behavioral assays was at least 2–3 days. Testing was conducted during the light cycle between 7 a.m. and 7 p.m. Equipment was thoroughly cleaned with a 0.1% acetic acid solution followed by thorough drying before each rat was tested. Rats were weighed after testing.

### 5.3. Activity Box

Novelty-seeking and habituation learning were assessed in an activity box, a small open field, as described previously [102]. The activity box (40 × 40 × 40 cm) was made of acrylic plastic and was equipped with an automated activity monitoring system (Tru Scan, Photobeam Sensor-E63-22; Coulbourn Instruments, Allentown, PA, USA). Activity box behavior was automatically monitored by means of two grids of infrared sensor beams mounted horizontally 2.5 cm and 14.5 cm above the floor for assessing distance travelled (in cm) and rearing behavior (number), respectively. The measure of rearing included all types of rearing, that is, irrespective of whether they were displayed on or off the walls. Testing began by placing the rat into a corner of the activity box, facing a wall. Activity box behavior was tested under red light (28 lx) conditions for 10 min on two consecutive days. Two and three rats were excluded from data analysis for the first and second day, respectively, due to data loss.

### 5.4. Elevated Plus Maze

Anxiety-related behavior was evaluated in an elevated plus maze, as previously described [102]. The apparatus was made of gray plastic and consisted of two opposed open arms and two opposed closed arms (arm sizes: 50 × 10 cm) extending from an open central square (10 × 10 cm). The maze was elevated 50 cm above the floor. Testing began by placing the rat into the center of the maze facing one of the open arms. Anxiety-related behavior was measured under conditions of white light (30 lx in the center) and videotaped using a digital camera (TVVR3304; ABUS, Affing, Germany). As parameter indicating anxiety-like behavior, time spent on open arms was analyzed using automated tracking software (Ethovison XT 14; Noldus, Wagenigen, The Netherlands). An open arm entry was defined as entry of the rat with all four paws including tail base. Overall locomotor activity was measured by means of distance travelled on the apparatus. Each rat was tested for 5 min on two consecutive days.

### 5.5. Novel Object Recognition

For assessing cognitive functioning, the novel object recognition test was conducted in a large open field made of gray plastic (60 × 60 × 60 cm), as previously described [103]. First, rats were habituated to the open field (no objects present) by placing them into the box for 10 min. Next, 24 h after the habituation session, the novel object recognition test was conducted, which consisted of three phases: acquisition trial, inter-trial interval, and recognition trial. In the acquisition trial, each rat was allowed to freely explore the open field containing two identical sample objects for 5 min. The objects were placed in one of the back corners of the box, with the objects situated 15 cm away from the walls. As objects, either two silver iron cylinders (5 cm in diameter, 8 cm high) or two red metal cubes (5 × 5 × 8 cm) were used in a counter-balanced manner. After the acquisition trial, the rats were returned to their home cages for 30 min, the inter-trial interval. During that time, one clean familiar object and one clean novel object were placed in the open field, where the two identical objects had been located during in the acquisition trial. After the inter-trial interval, each rat was returned to the open field for a 5 min recognition trial and allowed to freely explore the familiar and the novel object. For behavioral analyses, a digital camera (TVVR3304; ABUS, Affing, Germany) was mounted 1.5 m above the floor of the open field and connected to a personal computer for recording and data storage. Behavior was scored from video recordings by an experienced observer blind to the rat’s genotype using The Observer XT (Noldus, Wagenigen, The Netherlands). Object exploration was quantified as time spent sniffing the object and scored whenever the nose was oriented toward the object and the nose-object or front paw-object distance was 2 cm or less. Recognition memory was defined as spending more time sniffing the novel object than the familiar object. Testing was performed under white light (40 lx) conditions.

### 5.6. Differential Fear Conditioning

#### 5.6.1. Setup and Paradigm

Differential fear conditioning took place in a shock chamber (33.5 × 35 × 38 cm) made of gray and transparent plastic walls. The roof and one wall were made of transparent plastic to allow video observation during the test. A loudspeaker was mounted in one wall ~30 cm above the floor for presenting tones. The floor of the shock chamber was made of stainless-steel rods (diameter: 5 mm) spaced 1 cm apart. The chamber was placed in a sound attenuating isolation cubicle (51 × 71 × 51 cm; Coulbourn Instruments, Allentown, PA, USA) equipped with two white-light LED spots (~40 lx; Conrad Electronic, Hirschau, Germany) and a b/w CCD camera (Conrad Electronic, Hirschau, Germany) connected to a computer for videotaping. An UltraSoundGate Condenser CM 16 Microphone (Avisoft Bioacoustics, Berlin, Germany) was attached to the roof of the shock chamber ~30 cm above the floor. The microphone was connected via an UltraSoundGate 416 USB audio device to a computer, where acoustic data were recorded with a sampling rate of 250,000 Hz in 16-bit format (recording range: 0–125 kHz) by Avisoft RECORDER (Avisoft Bioacoustics, Berlin, Germany). The microphone is sensitive to frequencies of 15–180 kHz with a flat frequency response (±6 dB) between 25 and 140 kHz. 

The differential fear conditioning paradigm consisted of three test phases: acquisition, extinction, and recovery. On the first day, day 1, acquisition was performed. The next day, day 2, extinction was tested. One week later, day 9, recovery was measured. Two distinct contexts were used. Context A was defined by lavender scent (0.2% solution; Primavera Life GmbH, Oy-Mittelberg, Germany) placed underneath the stainless-steel rods and visual cues made of 3.8 cm broad vertical white stripes (Tesa SE, Norderstedt, Germany) with 3.8 cm distance in between. Context B was defined by lemongrass scent (0.2 % solution; Primavera Life GmbH, Oy-Mittelberg, Germany) and visual cues made of 3.8 cm broad horizontal white stripes (Tesa SE, Norderstedt, Germany) with 3.8 cm distance in between. Rats were habituated to the contexts for 300 s.

Acquisition was performed in context A. During acquisition, rats were trained to associate an acoustic stimulus (conditioned stimulus plus, CS+) with electric shock (unconditioned stimulus, UCS), whereas a second stimulus was never paired with electric shock (conditioned stimulus minus, CS-). As CS, 2 kHz and 9 kHz sinewave tones (generated with: Avisoft SASLab Pro Synthesizer) were presented at 72 dB for 30 s. As USC, a 0.5 mA scrambled shock (52 Hz, 120 V peak-to-peak amplitude; stand-alone shocker; Med Associates, St. Albans, USA) was used. The CS+ presentation was terminated by the electric shock of 500 ms duration. The CS- presentation was not terminated by an electric shock. Six presentations of CS+ and CS- each were applied in a pseudo-randomized, counter-balanced manner. Stimulus delivery and timing were controlled by the Presentation program (Neurobehavioral Systems, Albany, VT, USA). For acquisition, the rats were handled with gloves and carried to the testing apparatus on the arm of the experimenter.

Extinction was tested in context B the next day after acquisition. CS presentation (CS+/CS-) was pseudo-randomized and altered in comparison to acquisition. For extinction, the rats were handled without gloves and carried to the testing apparatus in a Macrolon Type II cage (27 × 22 × 14 cm).

Recovery was measured in context B on the seventh day after extinction. CS presentation (CS+/CS-) was pseudo-randomized and altered in comparison to acquisition and extinction. For recovery, the rats were handled identical to extinction, without gloves and carried to the testing apparatus in a Macrolon Type II cage (27 × 22 × 14 cm).

#### 5.6.2. Analysis of Immobility

Immobility was scored in 30 s time bins from video recordings by an experienced observer blind to the rat’s genotype using The Observer XT (Noldus, Wagenigen, The Netherlands), as previously described [37]. Immobility was defined as the suppression of all somatic motility except of motions associated with respiratory activity.

#### 5.6.3. Analysis of Ultrasonic Vocalizations

The emission of 22-kHz USV was analyzed by an experienced observer blind to the rat’s genotype using Avisoft SASLab Pro (Version 5.2.09; Avisoft Bioacoustics, Berlin, Germany), as previously described [37]. For acoustical analysis, high-resolution spectrograms (frequency resolution: 488 Hz; time resolution: 0.512 ms) were obtained through a fast Fourier transformation (512 FFT length, 100 % frame, Hamming window and 75% time window overlap). A lower-cut-off-frequency of 18 kHz was used to reduce background noise outside the relevant frequency band to 0 dB. Detection of 22-kHz USV was provided by an automatic threshold-based algorithm (threshold: −40 dB) and a hold time mechanism (hold time: 20 ms). Accuracy of 22-kHz USV detection was verified and a 100% concordance between automatic and observational detection was obtained. Total calling time was measured for entire test phases and separately for presentations of CS+ and CS-. As 22-kHz USV are emitted either as single pulses or in short bouts, calls were divided into those starting a bout versus those within a bout. Number of calls starting a bout and calls per bout were assessed.

### 5.7. Hot Plate

After differential fear conditioning, a hot plate test was performed under white light (~300 lx) to assess the effects of SERT deficiency on pain reactivity to thermal stimulation (precision hot plate, Prezitherm PZ35; Harry Geistigkeit GmbH, Düsseldorf, Germany). On the first day, the rat was placed onto the unheated apparatus for 120 s to habituate it to the test environment. On the next day, the rat was placed into the center of the hot plate kept at a constant temperature of 52 °C. The time to lick one of the four paws was measured by observation. To prevent tissue damage, a cut-off latency of 30 s was applied.

### 5.8. Statistical Analysis

All statistical tests were carried out using IBM SPSS Statistics (Version 25.0.0.1) software. To compare the body weight throughout different stages of the experiment, a repeated measures three-way ANOVA with the between-subject factors genotype (G) and sex (S) and the within-subject factor testing procedure was calculated, followed by repeated measures two-way ANOVAs separately for both sexes. 

To assess differences in the prevalence of rats emitting 22-kHz USV during differential fear conditioning between experimental conditions, chi^2^-tests were calculated. Overall immobility and 22-kHz USV emission were compared using two-way ANOVAs with the between-subject factors G and S. To determine differences in the reaction to CS+ and CS- presentations for the phases of extinction (EXT) and recovery (REC), repeated measures three-way ANOVAs with the between-subject factors G and S and the within-subject factor CS presentation (CS) were performed. The time course of immobility towards CS+ and CS- presentations was compared using a repeated measures four-way ANOVA with between-subject factors G and S and the within-subject factors of trial (TRIAL) in addition to CS. Single CS presentations at the beginning and the end of testing were compared using paired *t*-tests. As for the analysis of immobility throughout different days, the last CS presentations during EXT and the first during REC, respectively, were compared using a repeated measures four-way ANOVA with between-subject factors G and S and the within-subject factors of day (DAY) and CS. 

Novelty-seeking in the activity box, anxiety-related behavior in the elevated plus maze, and pain sensitivity in the hot plate test were compared using two-way ANOVAs with the between-subject factors G and S. Habituation learning in the activity box was compared using a repeated measures three-way ANOVA with between-subject factors G and S and the within-subject factor DAY. Cognitive functioning in the novel object recognition test was analyzed with a repeated measures three-way ANOVA with between-subject factors G and S and the within-subject factor of object (OBJ), i.e., percentage of exploration time for familiar versus novel object. Paired *t*-tests were calculated to compare the percentage of exploration time for familiar versus novel object in the different experimental conditions. To correlate immobility displayed during differential fear conditioning, including acquisition, extinction, and recovery, with novelty-seeking, anxiety-related behavior, habituation learning, cognitive functioning, and pain sensitivity, Pearson correlation coefficients were calculated.

Since sphericity was not met for several repeated-measures ANOVAs, Greenhouse-Geißer corrected values are reported. ANOVAs were followed by post-hoc LSD tests when appropriate, i.e., following significant ANOVA results. Two-tailed significance threshold was set at 5%. All values are reported as mean and ±standard error of mean (SEM).

## Data Availability

All data are available in the manuscript or supplementary materials.

## Supplementary Materials

The following are available online at https://www.mdpi.com/article/10.3390/ijms22137088/s1, Figure S1: CS+/CS- Presentation: immobility, last extinction vs. first recovery, grouped by genotype; Figure S2: CS+/CS- Presentation: 22-kHz USV, all rats; Figure S3: CS+/CS- Presentation: 22-kHz USV, grouped by genotype; Figure S4: novel object recognition. Figure S5: correlation, immobility, and 22-kHz USV.
