# Supplementary material for: Fear Extinction and Predictive Trait-Like Inter-Individual Differences in Rats Lacking the Serotonin Transporter
Source: Int J Mol Sci. 2021 Jun 30;22(13):7088. doi: 10.3390/ijms22137088 (PMC8268876; doi:10.3390/ijms22137088)
Supplement: Supplementary file 1 [file ijms-22-07088-s001.zip › ijms-1248782-supplementary.pdf]

Supplementary figures

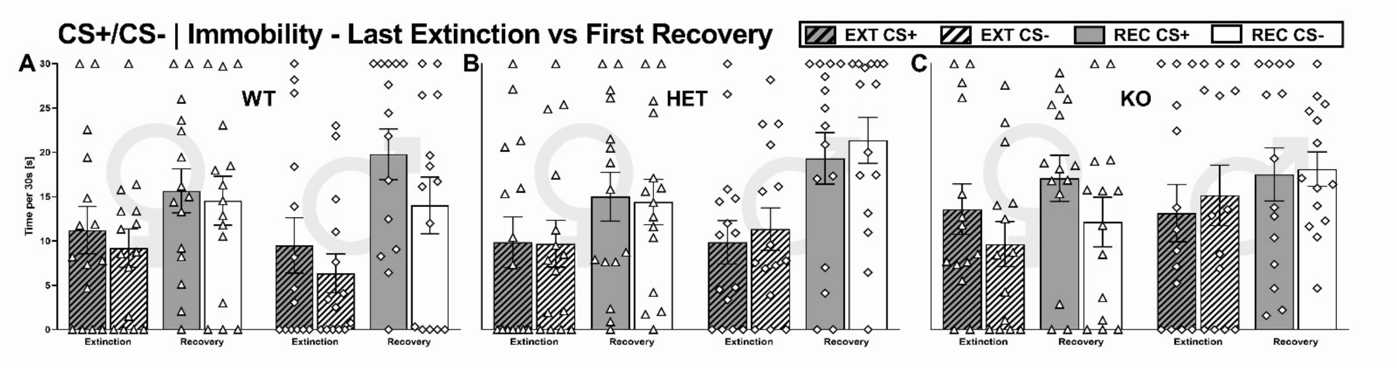

Figure S1. CS+/CS- Presentation | Immobility | Last Extinction vs First Recovery | Grouped by Genotype. Effects of SERT deficiency on immobility for the comparison of last trial extinction vs first trial recovery. Depicted are total amounts of immobility for last CS+ presentation (grey striped bar) and CS- presentation (white striped bar) during extinction (A, C), as well as CS+ presentation (grey bar) and CS- presentation (white bar) during recovery for $SERT^{+/+}$ (A), $SERT^{+/-}$ (B) and $SERT^{-/-}$ (C) rats. Two bars on the left comprise CS+ and CS- presentations for females; males are shown on the two right bars of every genotype. N = 29 $SERT^{+/+}$ (15 female, 14 male), 30 $SERT^{+/-}$ (15 female, 15 male), 28 $SERT^{-/-}$ (14 female, 14 male) rats. Data are presented as mean ± SEM.

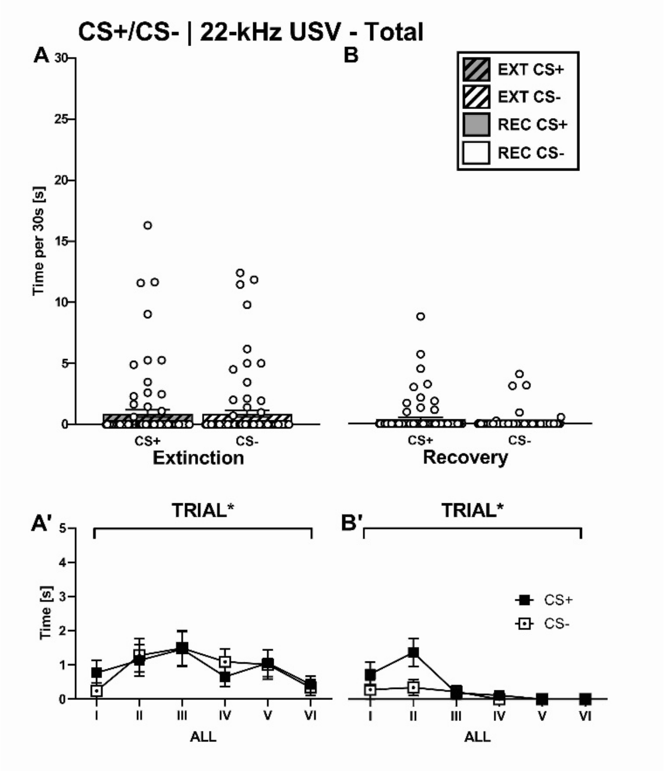

Figure S2. CS+/CS- Presentation | 22-kHz USV | All Rats. Effects of SERT deficiency on 22-kHz USV for CS+ and CS- presentations during extinction (A-A') and recovery (B-B'). Depicted are total amounts of 22-kHz USV for CS+ presentations (grey striped bar) and CS- presentations (white striped bar) during extinction (A), as well as CS+ presentations (grey bar) and CS- presentations (white bar) during recovery (B). Furthermore, single trial immobility levels for extinction (A') and recovery (B') are shown by means of CS+ presentations (black squares) and CS- presentations (white squares with dot). N = 87 rats. Data are presented as mean ± SEM. TRIAL* $p < 0.05$ effect of time

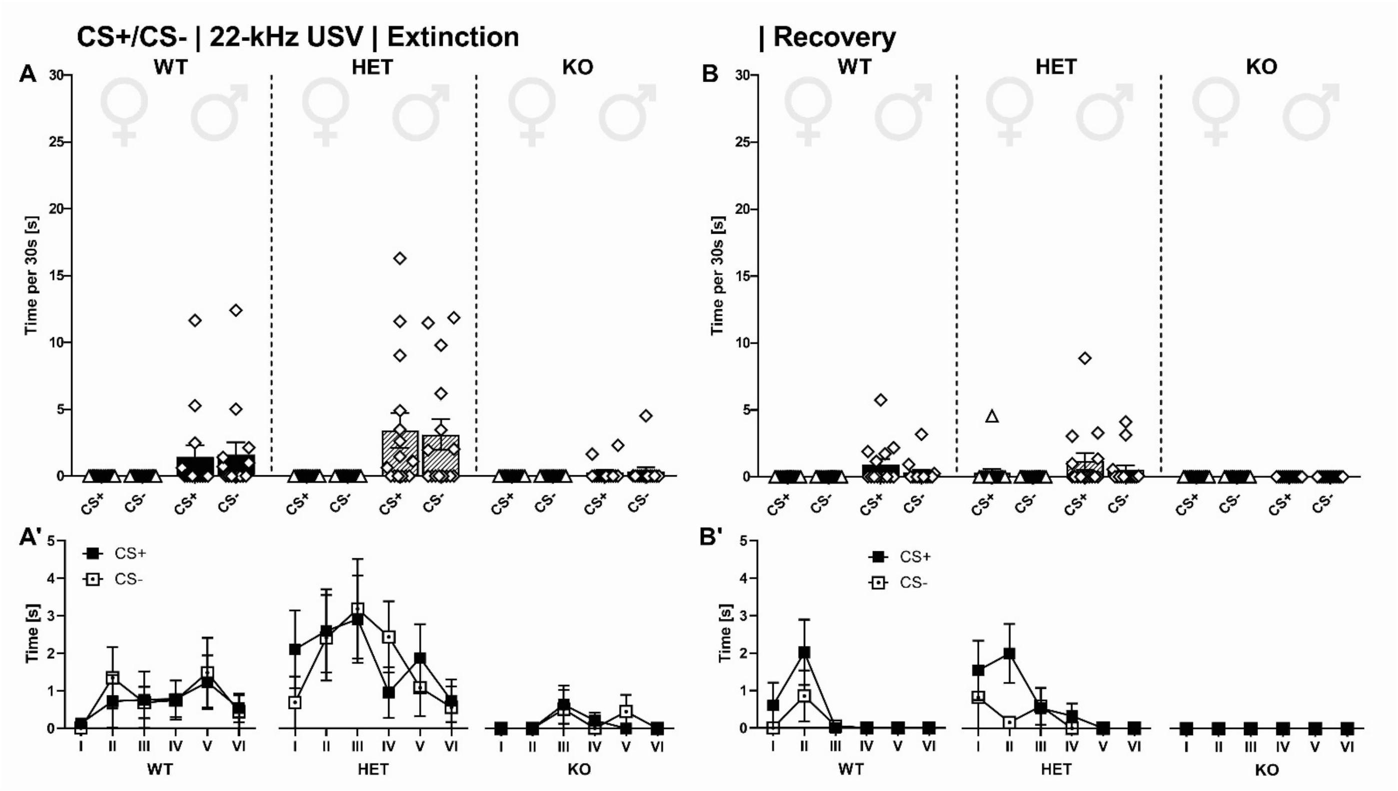

Figure S3. CS+/CS- Presentation ǀ 22-kHz USV ǀ Grouped by Genotype. Effects of SERT deficiency on 22-kHz for CS+ and CS- presentations during extinction (A-A') and recovery (B-B'). Depicted are total amounts of 22-kHz USV for both CS+ and CS- presentations for SERT$^{+/+}$ (black bar), SERT$^{+/-}$ (striped bar) and SERT$^{-/-}$ (white bar) rats. Two bars on the left comprise CS+ and CS- presentations for females; males are shown on the two right bars of every genotype. Furthermore, single trial immobility levels for extinction (A') and recovery (B') are shown by means of CS+ presentations (black squares) and CS- presentations (white squares with dot). N = 29 SERT$^{+/+}$ (15 female, 14 male), 30 SERT$^{+/-}$ (15 female, 15 male), 28 SERT$^{-/-}$ (14 female, 14 male) rats. Data are presented as mean ± SEM.

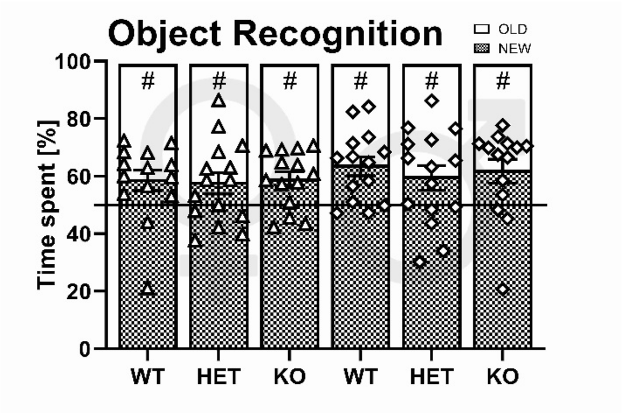

Figure S4. Novel Object Recognition. Effects of SERT deficiency on cognitive functioning. Depicted is percentage of time spent exploring a familiar object (old, white bar) vs a novel object (new, checkered bar) for SERT$^{+/+}$, SERT$^{+/-}$ and SERT$^{-/-}$ female rats (three left panels) and their male conspecifics (three right panels). N = 44 female rats (15 +/+, 15 +/-, 14 -/-), N=43 male rats (14 +/+, 15 +/-, 14 +/-). Data are presented as mean ± SEM. # $p < 0.05$ significant within-subject comparison of familiar vs novel object

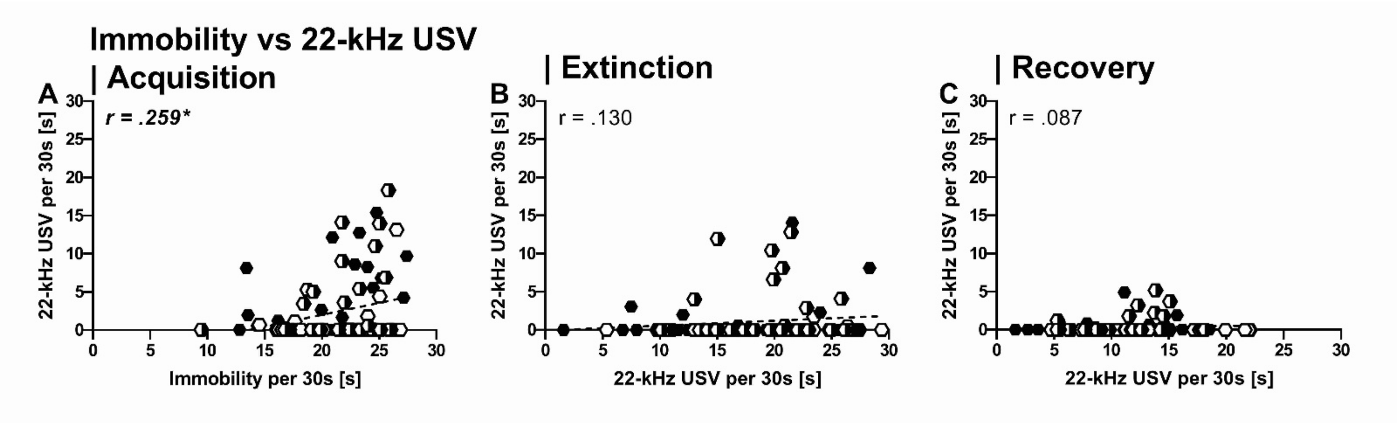

Figure S5. Correlation – Immobility and 22-kHz USV. Duration of immobility in relation to 22-kHz USV during acquisition (A), extinction (B), and recovery (C). Depicted are SERT$^{+/+}$ (black diamond), SERT$^{+/-}$ (black-white diamond), and SERT$^{-/-}$ (white diamond) rats. N = 29 SERT$^{+/+}$ (15 female, 14 male), 30 SERT$^{+/-}$ (15 female, 15 male), 28 SERT$^{-/-}$ (14 female, 14 male) rats. Data are presented as individual values and correlation coefficients. Statistical significance ($p < .05$) of correlation coefficient is indicated in ***bold and italic***.
